# Cooperation of the Inducible Nitric Oxide Synthase and Cytochrome P450 1A1 in Mediating Lung Inflammation and Mutagenicity Induced by Diesel Exhaust Particles

**DOI:** 10.1289/ehp.9063

**Published:** 2006-05-30

**Authors:** Hongwen Zhao, Mark W. Barger, Joseph K.H. Ma, Vincent Castranova, Jane Y.C. Ma

**Affiliations:** 1 Institute of Respiratory Diseases, First Affiliated Hospital, China Medical University, Shenyang, People’s Republic of China; 2 Health Effects Laboratory Division, National Institute for Occupational Safety and Health, Morgantown, West Virginia, USA; 3 School of Pharmacy, West Virginia University, Morgantown, West Virginia, USA

**Keywords:** cytochrome P450 1A1, cytokine production, diesel exhaust particles, inflammation, mutagenicity, nitric oxide

## Abstract

Diesel exhaust particles (DEPs) have been shown to activate oxidant generation by alveolar macrophages (AMs), alter xenobiotic metabolic pathways, and modify the balance of pro-antiinflammatory cytokines. In this study we investigated the role of nitric oxide (NO) in DEP-mediated and DEP organic extract (DEPE)-mediated inflammatory responses and evaluated the interaction of inducible NO synthase (iNOS) and cytochrome P450 1A1 (CYP1A1). Male Sprague-Dawley rats were intratracheally (IT) instilled with saline, DEPs (35 mg/kg), or DEPEs (equivalent to 35 mg DEP/kg), with or without further treatment with an iNOS inhibitor, aminoguanidine (AG; 100 mg/kg), by intraperitoneal injection 30 min before and 3, 6, and 9 hr after IT exposure. At 1 day postexposure, both DEPs and DEPEs induced iNOS expression and NO production by AMs. AG significantly lowered DEP- and DEPE-induced iNOS activity but not the protein level while attenuating DEPE- but not DEP-mediated pulmonary inflammation, airway damage, and oxidant generation by AMs. DEP or DEPE exposure resulted in elevated secretion of both interleukin (IL)-12 and IL-10 by AMs. AG significantly reduced DEP- and DEPE-activated AMs in IL-12 production. In comparison, AG inhibited IL-10 production by DEPE-exposed AMs but markedly increased its production by DEP-exposed AMs, suggesting that NO differentially regulates the pro- and antiinflammatory cytokine balance in the lung. Both DEPs and DEPEs induced CYP1A1 expression. AG strongly inhibited CYP1A1 activity and lung S9 activity-dependent 2-aminoanthracene mutagenicity. These studies show that NO plays a major role in DEPE-induced lung inflammation and CYP-dependent mutagen activation but a lesser role in particulate-induced inflammatory damage.

Epidemiologic studies have shown a correlation between exposure to ambient air particulate matter and adverse health outcomes manifested as an increased incidence of cardiovascular and respiratory mortality and morbidity (Dockery et al. 1993). Diesel exhaust particles (DEPs) are common airborne particulate matter that have diameters of < 2.5 μm and contain adsorbed organic compounds, many of which are known to alter the pulmonary microsomal enzyme pool, which results in altered xenobiotic metabolism. Studies in rats have shown that DEPs induce enhanced responses to allergic sensitization and weaken host defenses against bacterial infection through particulate- and/or organic component-mediated cellular responses (Takano et al. 2002; Yang et al. 2001; Yin et al. 2002, 2003).

Alveolar macrophages (AMs), through phagocytosis, release reactive oxygen species (ROS), reactive nitrogen species, and pro-inflammatory cytokines and are the principal cell type in the lung that mediates immune/ inflammatory responses against inhaled particles, chemicals, and microorganisms. The generation of superoxide anion by AMs through NADPH oxidase during the respiratory burst is important in eliminating extracellular pathogens (Segal 1989), whereas nitric oxide (NO) exhibits antimicrobial activity against pathogens that survive and proliferate in the intracellular environment (Takano et al. 1999). In human *Mycobacterium tuberculosis* infection, most tubercle bacilli reside in macrophages and generate inducible NO synthase (iNOS) expression as the cell’s major bactericidal activity (Choi et al. 2002). The organic content of DEPs varies with varying sources of DEPs and is known to modulate DEP toxicity (Singh et al. 2004). Exposure of cells to DEP organic extracts (DEPEs) has been shown to induce intracellular ROS generation, apoptosis (Hiura et al. 1999; Kumagai et al. 1997), and mutagenicity (DeMarini et al. 2004). A study of *Listeria monocytogenes* infection in the rat lung showed that DEPs suppress host clearance of *Listeria* through decreased ROS and NO generation, phagocytosis, and proinflammatory cytokine secretion by *Listeria*-activated AMs (Yang et al. 2001; Yin et al. 2002, 2003). DEPEs also induce ROS generation through the cytochrome P450 (CYP) monooxygenase system during the catalytic cycle (Puntarulo and Cederbaum 1998). CYP1A1, which is inducible by polycyclic aromatic hydrocarbons (PAHs) (Kuljukka-Rabb et al. 2001) present in DEPs, is known to induce oxidative stress and transforms procarcinogenic compounds to carcinogenic metabolites (Bondy and Naderi 1994). The normal rat lung contains CYP2B1 as the constitutive CYP isoform but has very low levels of CYP1A1 (Voigt et al. 1990). Exposure of rats to DEPs results in a significant and transient increase in CYP1A1 but a sustained decrease in CYP2B1, along with a suppression of the phase II enzymes, glutathione *S*-transferase (GST), and catalase (Rengasamy et al. 2003). Thus, DEP exposure can alter not only oxidant generation but also the metabolic activity–dependent mutagen activation in the lung (Zhao et al. 2004).

Our studies have shown that DEPs induce iNOS and NO production by naive AMs but inhibit both lipopolysaccharide (LPS)-mediated and *Listeria*-mediated NO production by rat AM (Yang et al. 1999, 2001), suggesting that DEPs may affect NO production directly as well as involving other mediators. It is well known (White and Marletta 1992) that iNOS is a hemoprotein that contains both a reductase and a heme domain on the same polypeptide. The flavin-containing reductase domain is similar in function to NADPH CYP reductase and is capable of producing superoxide with compounds such as quinones and nitroarenes found in DEPs (Kumagai et al. 1998). A concurrent production of NO and superoxide by iNOS may lead to the formation of peroxy-nitrite, a reactive intermediate that is known to cause protein damage through nitration of tyrosine, tryptophan, or cysteine residues (Lin et al. 2003). The fact that DEPs contain compounds that produce superoxide through interaction with the reductase domain of iNOS suggests that the production of NO and peroxynitrite may play a role in DEP-mediated pulmonary toxicity, including weakening of the lung’s host defense against bacterial infection.

Studies have suggested that NO may down-regulate certain CYP enzymes through interaction with the heme site. The inhibition of phenobarbital-induced CYP2B1/2 activity by LPS, for example, was attributed to NO-or peroxynitrite-mediated protein nitration (Khatsenko et al. 1997) at the Tyr190 residue (Lin et al. 2003). However, the effect of NO on the PAH-induced CYP1A1 activity, which is one of the more active CYP isoforms in superoxide generation (Puntarulo and Cederbaum 1998), has not been demonstrated. We hypothesized that NO plays an important role in modulating pulmonary inflammatory responses through an oxidant-mediated pathway and may also mediate CYP1A1 activity. In the present study, through *in vivo* inhibition of iNOS activity using aminoguanidine (AG), a selective inhibitor for iNOS (Misko et al. 1993), we examined a possible cooperative action between iNOS and CYP1A1 in mediating DEP-induced pulmonary inflammatory and mutagenic responses and investigated the roles of DEPEs and the particulate in mediating DEP-induced pulmonary toxicity.

## Materials and Methods

### Animal treatment

DEPs and DEPEs (National Institute of Standards and Technology, Gaithersburg, MD; Standard Reference Materials 2975 and 1975, respectively) were autoclaved and mixed with pyrogen-free sterile saline. The suspensions were sonicated for 5 min using an ultrasonic processor with a micro tip (Branson Sonifier 450; Branson Ultrasonics, Danbury, CT) before intratracheal (IT) instillation. We purchased specific pathogen-free male Sprague-Dawley (Hla:SD-CVF) rats (~ 200 g) from Hilltop Laboratories (Scottdale, PA). Rats were kept in cages individually ventilated with HEPA-filtered air, housed in a facility approved by the Association for Assessment and Accreditation of Laboratory Animal Care and provided food and water *ad libitum*. The animals were humanely treated and with regard for alleviation of suffering. Animals were used after a 1 week acclimatization period. Rats were anesthetized with sodium methohexital (35 mg/kg intraperitoneally) and placed on an inclined restraint board. A 0.3-mL suspension of DEPs at a dose of 35 mg/kg body weight, or equivalent amount of DEPEs contained in the DEP dose, or saline (control) was intratracheally instilled. We treated another group of rats with AG (100 mg/kg) by intraperitoneal injection at 30 min before and 3, 6 and 9 hr after IT instillation of DEPs, DEPEs, or saline. Rats were sacrificed at 1 day postexposure.

### Isolation of AMs and AM cultures

Animals were anesthetized with sodium pentobarbital (0.2 g/kg) and exsanguinated by cutting the renal artery. We obtained AMs by bronchoalveolar lavage with a Ca^2+^/Mg^2+^-free phosphate-buffered medium (145 mM NaCl, 5 mM KCl, 1.9 mM NaH_2_PO_4_, 9.35 mM Na_2_HPO_4_, and 5.5 mM glucose; pH 7.4) as described previously (Yang et al. 2001). The acellular supernate from the first lavage was saved separately from subsequent lavages for analysis of lactate dehydrogenase (LDH) activity and protein content. Cell pellets from each animal were centrifuged, combined, washed, and resuspended in a HEPES-buffered medium (145 mM NaCl, 5 mM KCl, 10 mM HEPES, 5.5 mM glucose, and 1.0 mM CaCl_2_; pH 7.4). Cell counts and purity were measured using an electronic cell counter equipped with a cell sizing attachment (Coulter model Multisizer II with a 256C channelizer; Coulter Electronics, Hialeah, FL).

AM-enriched cells were obtained by adherence of lavaged cells to the tissue culture plate as described previously (Yang et al. 1999) and cultured in fresh Eagle minimum essential medium (BioWhittaker, Walkersville, MD) for an additional 24 hr. AM-conditioned media were collected and centrifuged, and the supernates were saved in aliquots at −80°C for further analysis of cytokines.

### Determination of protein, LDH, and chemiluminescence

We monitored the acellular LDH activity in bronchoalveolar fluid (BALF) using Roche Diagnostic reagents and procedures on an automated Cobas MIRA PLUS analyzer (Roche Diagnostic Systems, Indianapolis, IN). We measured the protein content in the acellular BALF using a biuret reagent with Sigma diagnostic reagents and procedures (Sigma Chemical Co., St. Louis, MO) and following the manufacturer’s protocol. Chemiluminescence (CL) generated by AMs was determined using an automated luminometer (Berthold Autolumat LB 953; Wallac Inc., Gaithersburg, MD) as described by Yang et al. (2001). Briefly, CL generated by AMs was measured before and after stimulation with zymosan (2 mg/mL final concentration; Sigma Chemical Co.), a particle stimulant that activates macrophages. Zymosan-stimulated CL was calculated as the total counts in the presence of stimulant minus the corresponding basal counts.

### Cytokine assays and the determination of nitrite (NO_2_^−^) and peroxynitrite

Interleukin (IL)-12p40) and IL-10 in AM-conditioned media were determined using ELISAs (Biosource International Inc., Camarillo, CA) according to the manufacturer’s protocol. NO production was determined in AM-conditioned medium using the Greiss reaction (Green et al. 1982). We measured peroxynitrite by monitoring the formation of rhodamine 123 fluorometrically (500 nm excitation, 536 nm emission) according to the rhodamine standard curve (Kooy et al. 1994), using a PerkinElmer LS50 Luminescence Spectrometer (PerkinElmer, Inc., Norwalk, CT).

### Preparation of lung S9 and microsomal fractions

Rats from various exposure groups were anesthetized with pentobarbital sodium (0.2 g/kg), and the heart and lungs were removed. The lung S9 was obtained by centrifugation of tissue homogenate at 9,000 × *g* for 20 min at 4°C; the supernatant was saved as lung S9 fraction as described previously (Zhao et al. 2004) and stored at −80°C until use. The microsomal (pellet) and cytosolic (supernatant) fractions of the tissue homogenate were obtained by differential centrifugation. We then resuspended the microsomal pellet in incubation medium at a tissue concentration of 1 g/mL and determined protein concentrations of both fractions using a bicinchoninic acid protein assay kit (Pierce, Rockford, IL).

### Enzyme level and activity assay

At 1 day postexposure, CYP1A1 and CYP2B1 were fractionated by sodium dodecyl sulfate poly-acrylamide gel electrophoresis and transferred to a nitrocellulose membrane for Western blot analysis, using rabbit polyclonal antibodies specific for CYP1A1 or CYP2B1, to measure CYP1A1 and CYP2B1 levels in lung microsomes, as described previously (Rengasamy et al. 2003). We measured the activities of 7-ethoxyresorufin *O*-dealkylase (EROD) and 7-pentoxyresorufin *O*-dealkylase (PROD) by monitoring the production of resorufin fluorometrically according to the method of Burke et al. (1994) and using a luminescence spectrometer (model LS50; PerkinElmer, Inc.). We quantified the resorufin formation using a resorufin standard curve and measured the activity of GST by monitoring GST-dependent conjugation of glutathione and 1-chloro-2,4-dinitrobenzene spectrophotometrically using a model UV-2401 PC spectrophotometer (Shimadzu, Columbia, MD) at 340 nm (Habig et al. 1974). We measured the quinone reductase (QR) activity using a spectrophotometric assay in which we monitored the rate of reduction of 2,6-dichlorophenolindophenol at 590 nm with the spectrophotometer (Rengasamy et al. 2003). The catalase activity was determined using a catalase assay kit (Cayman Chemical Co., Ann Arbor, MI) according to manufacturer’s protocol.

### Salmonella typhimurium*/microsomal assay.*

We used *S. typhimurium* strain YG1024, which detects frameshift mutagens, for the Ames test and 2-aminoanthracene (2-AA; 0.015 μg/plate), a mutagen that requires metabolic activation, as the substrate. We performed the *Salmonella* mutation test using a modified microsuspension assay described in a previous study (Zhao et al. 2004). We performed all assays in duplicate plates and repeated each experiment at least 3 times with different animals. Spontaneous revertants were subtracted from the number of revertants obtained for each assay.

### Statistical analysis

Data are presented as means ± SEs. Comparisons were made using one-way analysis of variance with means testing by Dunnett’s test. *p*-Values < 0.05 were considered to be significant.

## Results

### Differentiation of DEP- or DEPE-induced iNOS expression and activity in AMs

**Figure 1:**
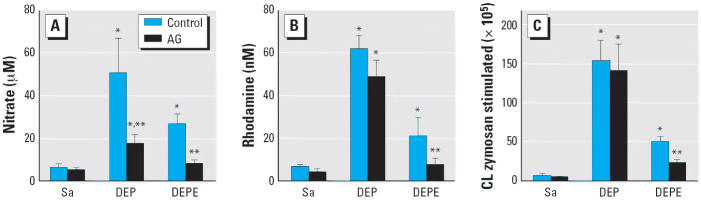
Effects of AG treatment on DEP- and DEPE-induced oxidant generation at 1 day postexposure: nitrite (*A*), peroxynitrite (*B*), and CL (*C*) production by AMs. Sa, saline. Results are presented as mean ± SE from at least six different animals. *Significantly different from control group, *p* < 0.05. ^**^Significantly different from the non-AG-treated group, *p* < 0.05.

Western blot analysis shows that both DEP and DEPE induced iNOS expression in AMs, with levels 12- and 6-fold above the control, respectively (data not shown). This induction of iNOS expression correlated with significant increases in cellular production of NO (8-fold for DEPs and 4-fold for DEPEs) and peroxynitrite (10-fold for DEPs and 3-fold for DEPEs) (Figure 1A,B). AG did not affect DEP- or DEPE-induced iNOS expression in AMs but significantly decreased DEP- or DEPE-induced NO production (Figure 1A). In contrast, AG significantly attenuated peroxynitrite production after DEPE exposure but not after exposure to DEPs (Figure 1B). In addition Figure 1C shows that the overall production of oxidants by DEP-exposed AMs in response to zymosan challenge was much greater than that of DEPE-exposed AMs, and that AG blocked oxidant generation resulting from DEPEs but not from DEPs. These results suggest that DEPs may induce oxidant generation through iNOS as well as particle-induced respiratory burst activity, but oxidants generated by DEPE-exposed AMs occur mainly through the intracellular iNOS pathway.

### Role of iNOS in DEP- and DEPE-induced lung injury and AM production of cytokines

**Figure 2:**
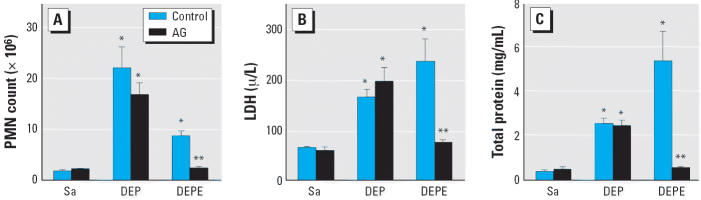
Role of NO in DEP- and DEPE-induced acute pulmonary inflammation, cellular toxicity, and lung injury. The first acellular lavage fluid of the bronchoalveolar lavage was used to assay for inflammatory or damage parameters. Sa, saline. Inflammation was determined by polymorphonuclear cell (PMN) infiltration (*A*), cytotoxicity was determined by monitoring LDH activity (*B*), and air–blood barrier damage was monitored as protein content (*C*) in the lavage fluid (*n* = 6–8). *Significantly different from control group, *p* < 0.05. ^**^Significantly different from the non-AG-treated group, *p* < 0.05.

Both DEP and DEPE exposures caused a significant neutrophil recruitment (Figure 2A), cytotoxicity measured as increased LDH activity in the lavage fluid (Figure 2B), and damage to the alveolar air–blood barrier as indicated by increased protein content in the lavage fluid (Figure 2C). Inhibition of NO production by AG attenuated the effect caused by DEPEs but not by DEPs. Together, Figures 1 and 2 suggest that DEP-induced acute lung injury involves particle-induced respiratory bursts, whereas DEPEs induce cytotoxicity through an intracellular mechanism that more strongly involves the expression and activity of iNOS. Thus, AG was not effective on particle-induced oxidant generation.

**Figure 3:**
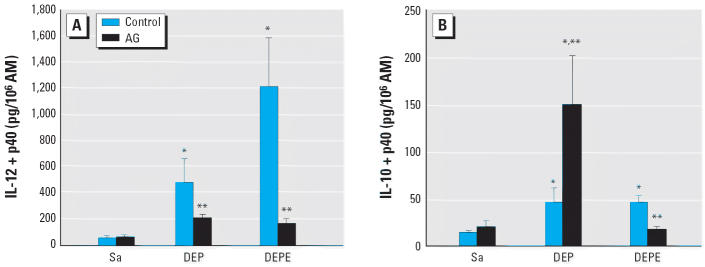
Role of NO in DEP- and DEPE-mediated pro- and antiinflammatory cytokine production by AM. Sa, saline. AM cells were isolated from different exposure groups with or without AG treatment. IL-12 (*A*) and IL-10 (*B*) production in the supernatant of the AM culture medium, at 37°C for 24 hr, was assayed using ELISA kits (*n* = 6–8). *Significantly different from control group, *p* < 0.05. ^**^Significantly different from the non-AG-treated group, *p* < 0.05.

AMs from both DEP- and DEPE-exposed rats secreted elevated levels of IL-12 and IL-10 compared with those of saline control rats (Figure 3). IL-12 is a proinflammatory cytokine known to elicit a T-lymphocyte–mediated immune response against bacterial infection (Hsieh et al. 1993), whereas IL-10 is an antiinflammatory cytokine known to prolong the survival of intracellular pathogens in AM (Redpath et al. 2001). AM production of IL-12 and IL-10 in response to DEPEs was markedly inhibited by the AG treatment, suggesting that the production of both cytokines is mediated through an NO-sensitive pathway. In comparison, AG treatment significantly decreased DEP-induced IL-12 production but further enhanced IL-10 release from AMs. This suggests that for DEP exposure, the role of iNOS in the production of IL-12 and IL-10 differs markedly.

### Effect of iNOS activity on DEP- or DEPE-induced expression and activity of CYP enzymes

**Figure 4:**
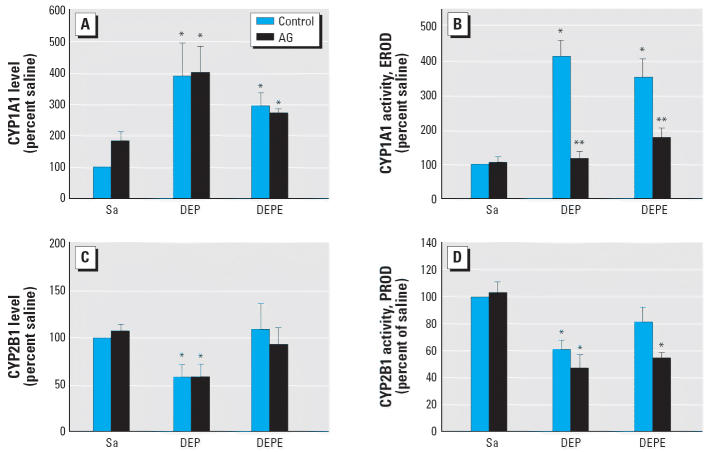
Effects of AG treatment on CYP1A1 and CYP2B1 after DEP or DEPE exposure. At 1 day postexposure, the intensities of CYP1A1 (*A*) and CYP2B1 (*C*) protein levels were quantified by densitometry and compared with the saline (Sa) control and are presented here as percentage of control. The activities of CYP1A1 and CYP2B1 were determined by monitoring EROD (*B*) and PROD (*D*) activity, respectively, and are presented as percentage of control. The control saline value for CYP1A1 was 2.34 ± 0.68 pmol/min/mg protein and for CYP2B1 was 5.24 ± 1.84 pmol/min/mg protein (*n* = 6–8). *Significantly different from control group, *p* < 0.05. ^**^Significantly different from the non-AG-treated group, *p* < 0.05.

**Figure 5:**
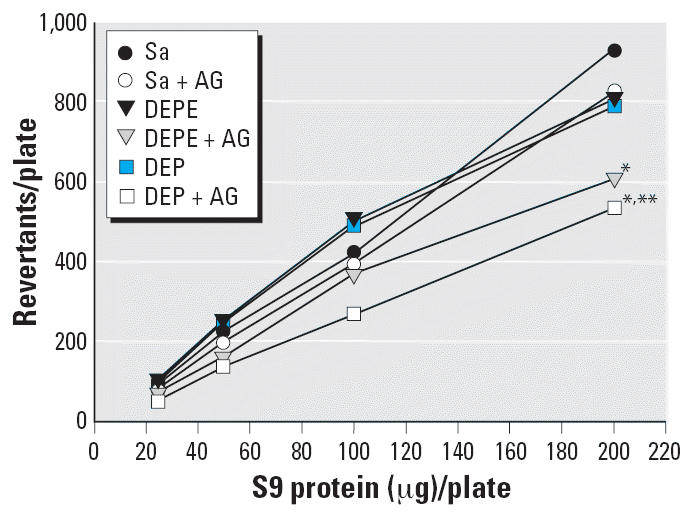
Effects of AG treatment on lung S9-dependent 2-AA mutagenicity in *S. typhimurium* strain YG1024. Sa, saline. Lung S9 were isolated at 1 day after saline, DEP, or DEPE exposure with or without AG treatment. For each sample, Ames assays were performed on duplicate plates. The spontaneous revertants (29 ± 4/plate) were subtracted from the number of revertants obtained for each assay. Results are presented as mean ± SE (*n* = 3). *Significantly different from control group, *p* < 0.05. ^**^Significantly different from the non-AG-treated group, *p* < 0.05.

The induction of CYP1A1 expression and activity in lung microsomes by DEPs and DEPEs at 1 day postexposure is shown in Figure 4. The AG treatment did not alter CYP1A1 protein levels in either DEP- or DEPE-exposed lung microsomes (Figure 4A) but markedly decreased CYP1A1 activity (Figure 4B). This suggests that the production of NO may be required for CYP1A1 activity. The results also show that exposure to DEPs but not to DEPEs significantly reduced CYP2B1 protein (Figure 4C) and PROD activity (Figure 4D) in the rat lung, and AG did not affect the expression or activity of CYP2B1 in either exposure system. DEPE exposure had no effect on cytosolic phase II enzymes (Table 1). However, DEP exposure reduced the activities of cytosolic GST and catalase, but not that of QR, and these DEP effects were not influenced by AG. These results further reveal a divergent effect of the particulate versus organic components of diesel exhaust, in that DEPEs induce CYP1A1, whereas the particulate reduces CYP2B1 and phase II metabolic enzymes. Figure 5 shows a positive involvement of CYP1A1 in and a clear effect of NO on the lung S9-dependent metabolic activation of 2-AA mutagenicity. AG, which attenuated CYP1A1 activity, caused a significant and consistent lowering of 2-AA mutagenic activation, suggesting that the production of NO is crucial for the activation of CYP1A1 activity and influences metabolic activation of mutagens.

## Discussion

DEPs alter both the acute and chronic immune/inflammatory responses and modify the outcome of such disease states as respiratory infection (Yang et al. 2001; Yin et al. 2002, 2003, 2004), allergic asthma (Nel et al. 2001), and lung cancer (Iwai et al. 2000). Our objective in the present study was to investigate the role of NO, induced by DEPs or DEPEs, in mediating the particle-induced and/or organic component–induced lung damage, cytokine production by AMs, the alteration of pulmonary xenobiotic metabolic pathways, and metabolic activity–mediated mutagenic activity. NO, a relatively stable uncharged radical that readily crosses lipid membranes, is a good inter- and intracellular trafficker in mediating many cellular responses (Crow 1997). Our studies show that with DEPE exposure, in the absence of particle core, NO is directly linked to lung inflammation and injury because inhibition of iNOS activity by AG abolished these adverse responses. DEP-induced inflammatory responses, however, were not significantly affected by AG treatment, which suggests that particulate-induced oxidant generation through the respiratory burst is responsible for lung injury. These results establish that both the particle core and DEPEs contributed to DEP-induced oxidative lung damage.

We have previously shown that exposure of rats to DEPs before *Listeria* infection significantly reduced *Listeria*-stimulated NO production by AMs, resulting in a slower intrapulmonary *Listeria* clearance (Yang et al. 2001). In the present study we show that both DEPs and DEPEs induce AMs in the production of the proinflammatory cytokine IL-12. This elevated IL-12 production is significantly diminished when rats were treated with AG, suggesting that NO is a key mediator that initiates IL-12 production, which may lead to IL-12-dependent T-lymphocyte–mediated immune responses. Conversely, both DEP and DEPE exposure caused elevated IL-10 production. The AG treatment, however, inhibited IL-10 release from DEPE-exposed AMs but enhanced IL-10 production from DEP-exposed AMs, suggesting that NO down-regulates IL-10 secretion in particle-exposed rats. This is consistent with our previous studies in which DEPs suppressed the immunity against *Listeria* by down-regulating IL-12 and up-regulating IL-10 production by *Listeria*-infected AM (Yin et al. 2004). These findings show that iNOS modulates the balance of AM-derived inflammatory mediators for the host defense against bacterial infection in DEP- or DEPE-exposed lung.

In the present study we show that the induction of CYP1A1 by exposure to DEPs or DEPEs was accompanied by an induction of iNOS and that inhibition of iNOS activity by AG nearly abolished CYP1A1 activity as demonstrated by EROD. In comparison, the constitutive CYP2B1 and cytosolic GST and catalases in the lung were reduced by DEPs but not by DEPEs and were not affected by AG treatment, suggesting that NO was not significantly involved in particle exposure–induced enzyme degradation. Studies of the role of NO in regulation of CYP enzymes have yielded contradicting results, with most studies suggesting that NO or peroxynitrite may down-regulate the CYP enzyme activity. Lin et al. (2003) showed that peroxynitrite can cause nitration of Tyr190 of CYP2B1 and lead to its inactivation. Studies have also suggested that NO, through LPS stimulation, suppressed CYP1A activity in astrocytes (Nicholson et al. 2004). In a Kupffer cell–hepatocyte co-culture, however, LPS markedly down-regulated hepatic phenobarbital-induced CYP2B1 activity through induced tumor necrosis factor-α released from Kupffer cells but not linked to NO production by either cell type (Milosevic et al. 1999). We demonstrate in the present study that DEP- or DEPE-stimulated NO production through iNOS plays a key role in activating CYP1A1 activity, as treating rats with AG inhibited iNOS activity and decreased CYP1A1 activity. Of interest, *ex vivo* addition of AG to isolated lung microsomes did not affect the EROD assay (data not shown), suggesting that AG does not directly interact with CYP1A1. DEPEs are known to contain substrates for NADPH CYP reductase that produce super-oxide and DNA scission (Kumagai et al. 1997). Our results show that exposure to DEPs or DEPEs can result in concurrent release of NO and ROS such as superoxide anion (Figure 1A,C) that lead to the production of peroxynitrite (Figure 1B), which may cause oxidative damage to proteins and DNA. The ratio of ROS to NO with respect to DEP exposure, as measured by CL, is greater than that for DEPE exposure because AG inhibited DEPE-induced but not DEP-induced CL. This suggests that iNOS plays a major role in the organic component–induced oxidant generation, whereas the induction of oxidant generation by DEPs may involve other mechanism(s) such as particle-induced macrophage respiratory bursts.

The potential carcinogenic effects of the particulate and organic components of DEPs remain unclear. There is a lack of correlation between carcinogenesis and the organic components of DEPs (Gallagher et al. 1994). Other studies have suggested that DEP-induced ROS generation may lead to DNA damage and the initiation of lung carcinogenesis (Ichinose et al. 1997; Iwai et al. 2000). In the present study we show that DEP exposure results in significant induction and/or inactivation of certain pulmonary phase I and phase II metabolic enzymes. The dose-dependent 2-AA activation by DEP- and DEPE-exposed S9 is similar to that of the control; however, the effect of DEP- or DEPE-exposed S9 on mutagen activation was significantly inhibited by AG, which suggests a cooperative reaction of iNOS and CYP1A1 activity. In addition, following phase I metabolism, xenobiotics are further metabolized or detoxified by phase II enzymes such as GST and QR, which have been shown to modify carcinogen metabolism and cancer susceptibility (Clapper 2000). Our study shows that through oxidant generation, DEPs can cause alteration of the metabolic pathways in the lung, including NO-mediated CYP1A1 activity and inactivation of CYP2B1 and cytosolic GST and catalase, and contribute to lung mutagenicity.

In summary, this study shows that iNOS, induced by DEPs and DEPEs, plays an important role in mediating DEP-induced pulmonary cellular responses. DEPE-induced NO, in the absence of particle core, causes pulmonary inflammation and lung damage and mediates the release of both IL-12 and IL-10 by AM. Conversely, NO is only partially involved in DEP-induced inflammation, which involves both the organic chemical effect and particle core–induced macrophage respiratory burst. NO is key in mediating the balance of pro- and antiinflammatory cytokines in the DEP-exposed lung. Enhanced production of IL-10 may increase the susceptibility of DEP-exposed lung to bacterial infection. Furthermore, NO was found necessary for CYP1A1 activity in both DEP- and DEPE-exposed lungs. Inhibition of NO by AG resulted in a lowered capability of the lung to activate metabolic-activity–dependent mutagenic agents.

## Figures and Tables

**Table 1:** Effects of AG treatment on DEP- or DEPE-exposed rat cytosolic phase II enzyme activities, GST, catalase, and QR.

	Percentage of saline control					
	GST		Catalase		QR	
Treatment	DEP	DEPE	DEP	DEPE	DEP	DEPE
None	79 ± 3*	88 ± 7	73 ± 3*	135 ± 22	87 ± 3	96 ± 13
AG treatment	82 ± 5*	109 ± 7	67 ± 11*	199 ± 16**	95 ± 10	85 ± 6

Rats were instilled IT with DEP or DEPE with or without AG treatment. The activities of GST, catalase, or QR were measured in the lung cytosol at 1 day postexposure as described in “Materials and Methods.” The activities of GST, catalase, and QR are expressed as the percent change relative to saline control. The specific activities in the saline control for GST, catalase, and QR were 204 ± 10, 80 ± 9 , and 659 ± 20 nmol/min/mg, respectively.

* Significantly different from the saline control, *p* < 0.05.

** Significantly different from the same exposure without AG treatment.
